# Does Milk Cause Constipation? A Crossover Dietary Trial

**DOI:** 10.3390/nu5010253

**Published:** 2013-01-22

**Authors:** Elesa T. Crowley, Lauren T. Williams, Tim K. Roberts, Richard H. Dunstan, Peter D. Jones

**Affiliations:** 1 Department of Rural Health NSW, University of Newcastle, Tamworth, NSW 2340, Australia; 2 Tamworth Rural Referral Hospital, Tamworth, NSW 2340, Australia; 3 Nutrition and Dietetics, University of Canberra, Bruce, ACT 2617, Australia; E-Mail: Lauren.williams@canberra.edu.au; 4 School of Health Sciences, University of Newcastle, Callaghan, NSW 2308, Australia; 5 School of Environmental and Life Sciences, University of Newcastle, Callaghan, NSW 2308, Australia; E-Mails: tim.roberts@newcastle.edu.au (T.K.R.); Hugh.dunstan@newcastle.edu.au (R.H.D.); 6 School of Medicine, Bond University, Robina, QLD 4226, Australia; E-Mail: pejones@bond.edu.au

**Keywords:** constipation, child, hypersensitivity, soy, casein

## Abstract

The aims of this study were to: (1) determine whether replacement of cow’s milk protein with soy resolves Chronic Functional Constipation (CFC); and (2) investigate the effects of cow’s milk β casein A1 and cow’s milk β casein A2 on CFC. Children diagnosed with CFC were recruited to one of two crossover trials: Trial 1 compared the effects of cow’s milk and soy milk; Trial 2 compared the effects of cow’s milk β casein A1 and cow’s milk β casein A2. Resolution of constipation was defined as greater than eight bowel motions during a two week intervention. Thirteen children (18 to 144 months) participated in Trial 1 (6 boys, 7 girls). Nine participants who completed the soy epoch all experienced resolution (*p* < 0.05). Thirty-nine children (21 to 144 months) participated in Trial 2 (25 boys, 14 girls). Resolution of constipation was highest during the washout epoch, 81%; followed by cow’s milk β casein A2, 79%; and cow’s milk β casein A1, 57%; however, the proportions did not differ statistically. The results of Trial 1 demonstrate an association between CFC and cow’s milk consumption but Trial 2 failed to show an effect from type of casein. Some other component in cow’s milk common to both A1 and A2 milk may be causing a problem in these susceptible children.

## 1. Introduction

Chronic functional constipation (CFC), defined as having one bowel motion every 3 to 15 days [1], occurs commonly in children. The frequency of CFC has been estimated to be as high as 36% of children who attend a consultation with a paediatrician [2]. A sensitivity to cow’s milk protein (CMP) has been proposed as a possible cause for CFC [3]. A potential link between CMP and constipation was first referred to in the allergy literature as early as the 1950’s [4]. Our systematic review of the literature from 1980 to 2008 assessing the evidence for a causal relationship between CMP intake and CFC in children found a small body of evidence which was suggestive rather than conclusive [5]. Randomised trials are needed to provide higher level evidence. 

Iacono and colleagues used a double-blind crossover trial design to compare the response to cow’s milk with that of soy milk in 65 children with chronic constipation [3]. Thirty three children were commenced on cow’s milk and 32 on soy milk for two weeks, followed by an unrestricted diet for one week, and then they were given the alternate milk for another two weeks. Constipation resolved (greater than 8 bowel motions per fortnight) for 68% of children consuming soy milk, but not for any of those consuming cow’s milk [3]. The researchers observed that children whose constipation resolved on the soy milk possessed a number of symptoms associated with cow’s milk protein allergy/intolerance at baseline, suggesting a pre-existing abnormality of the immune system. The main limitation of this study was the uncontrolled nature of the diet in between the two epochs. To date, the Iacono study has not been replicated by other researchers. The first stage of this current study, Trial 1, aimed to evaluate whether total removal of cow’s milk from the diet and substitution with soy milk, could resolve chronic constipation in children with CFC resistant to usual treatment in Australia.

The Iacono findings suggested that CMP consumption was a problem in these children, and proposed that the basis of CFC involved an allergic immune response to the ingested protein. Approximately 80% of total protein in cow’s milk is casein, and 30% to 35% of this is in the form of β-caseinwhich may contain an allergenic component [6]. β-Casein consists of a chain of 209 amino acids which can differ in structure depending on the breed of cowas a result of an adenine-cystosine substitution mutation giving β casein A2 translating in to a proline at position 67 rather than a histidine [7,8]. The β casein A1histidine variant allows enzymatic cleavage with production of a seven amino acid peptide called “β casomorphin 7” (BCM7) which has been shown to affect gut transit time [7,8]. It is hypothesised that in susceptible people, leakage of the peptide into the gut might result in CFC. In Trial 2, we aimed to investigate the effect of cow’s milk β casein A1 and cow’s milk β casein A2 on children with CFC resistant to traditional treatment.

## 2. Method

### 2.1. Study Design

**Figure 1:**
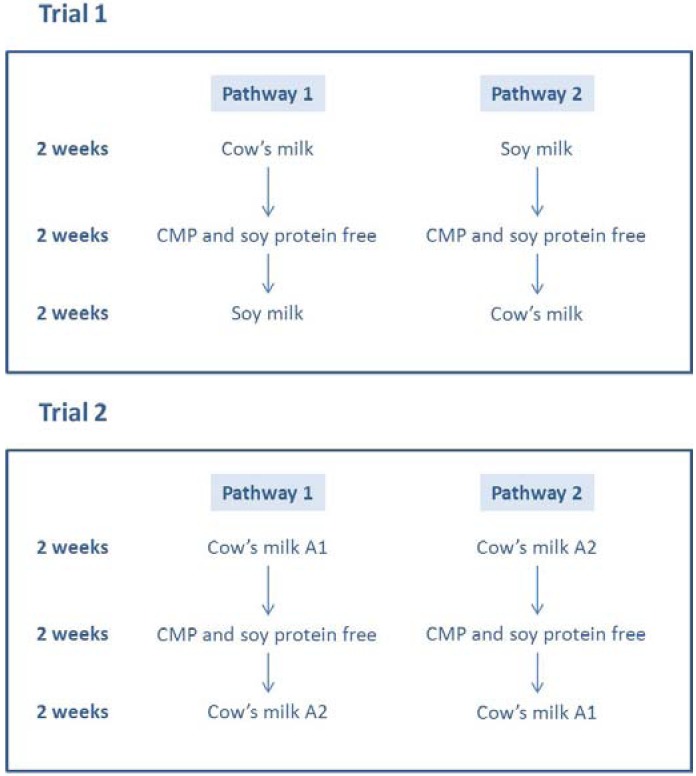
Trials and pathways to which participants were assigned in the cross-over study.

Two separate crossover clinical trials were conducted. Trial 1 compared the effect of cow’s milk and soy milk on CFC and was conducted in Tamworth, New South Wales. The second trial compared cow’s milk β casein A1 with cow’s milk β casein A2 and was conducted in Newcastle, New South Wales. With the exception of milk type, the study epochs were identical and the crossover design used in both trials is described below. As each child and their family consented to participate in the study they were assigned (according to whether the participants’ day of birth was odd or even), to pathway 1 or pathway 2 for each trial, by the research assistant (Figure 1). All researchers were blinded to the assignment. Ethical approval for the study was granted by Hunter New England Area Health Service Ethics Committee and the University of Newcastle Human Research Ethics Committee in 2005 (Reference 03/08/13/12/3.23). The aim was to recruit 30 children to Trial 1 and 40 children to Trial 2. The pathway of the study is shown in Figure 1.

### 2.2. Participants

Children (1–12 years) diagnosed by a paediatrician with CFC, defined as less than eight bowel motions per fortnight [3], unresolved by medications or diet, were recruited from medical and health services within the Hunter New England Region of New South Wales, from 2005 to 2007. Potential participants were made aware of the study by approved information sheets available in the surgery and via the clinicians. Families were provided with an information package about the study including: a participant information sheet; a sheet describing the milk free diet; and a reply-paid envelope. Participation in the study commenced when the researcher received a signed consent form. Children with Hirschsprungs disease, Cerebral Palsy, Coeliac disease and using medications known to cause constipation were excluded from the study. Children previously prescribed laxatives for chronic functional constipation withheld these for the duration of the trial. 

### 2.3. Protocol

**Figure 2:**
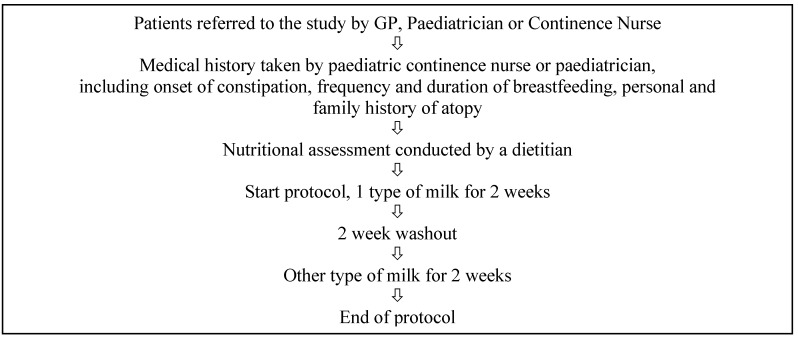
Flowchart of the trial protocol.

The trial procedure is shown in Figure 2. A detailed medical history was taken by the diagnosing paediatrician prior to commencing the trial. A detailed diet history, including amount of milk and dairy products usually consumed, was taken by the dietitian. Participants and/or their parents were supplied with the assigned milk for each of the two week trial periods and advised how to consume at least 400 mL of this milk each day and educated by a dietitian in how to avoid all other sources of CMP, without varying their usual fibre and fluid intake. The first two-week trial period was followed by a two-week washout period to remove possible confounders. The washout period was free of soy and cow’s milk protein, β casein A1 and cow’s milk β casein A2. Participants were encouraged to consume an additional 400 mL of other fluid during the washout period in place of the intervention milk to prevent dehydration. They were offered calcium supplementation during this period. After a two-week washout period, participants were switched to the alternate milk epoch for another two weeks. Researchers were unaware of the order of treatment and milk in commercial containers was rewrapped by the research assistant to promote double blinding. 

### 2.4. Primary Outcome Measures

#### 2.4.1. Bowel Movements

The average number of bowel motions per fortnight, prior to commencement of the study, was checked to assess eligibility and noted. A “Constipation Study Patient Diary” using the Bristol Stool Scale [9], previously validated against gut transit time [10,11,12], was kept by participants or their parents for the duration of the six week study. Records were kept of the number of bowel movements per day; whether straining was required to pass the motion; stool form and appearance in comparison to diagrams on the back of the diary; and symptoms of abdominal pain/discomfort and bloating. A clinical outcome was defined as eight or more bowel movements during a two week treatment period [3].

#### 2.4.2. Other Participant Data

Data collected included: onset, symptoms and duration of the constipation; medication history; frequency and duration of breast feeding; personal and family history of allergy and intolerance. 

### 2.5. Statistical Analysis

The statistics package *SPSS*™ version 16.0 was used for analysis. A linear mixed method analysis of the data was conducted to determine the effect of the treatments for the numeric outcome variables. Repeated measures analysis of variance was used to compare each of the three milk epochs; In Trial 1: soy milk, milk free and cow’s milk; In Trial 2: cow’s milk β casein A1, milk free and cow’s milk β casein A2. Verification of this analysis was conducted using contrasts and pair wise comparisons. Categorical versions of the numeric variables were used to statistically compare the number of bowel motions during the three two week trial periods for each milk epoch. The McNemar test for paired observations was used. Continuous variables were tested for normality then paired *t*-tests and non-parametric tests used to compare the number of bowel motions per participant per fortnight on each of the milk epochs. The Friedman test and Wilcoxon Ranked Sign were used to verify analysis conducted by the parametric tests.

## 3. Results

### 3.1. Participant Characteristics at Baseline

Fourteen children were initially recruited to Trial 1, with one later excluded due to a subsequent diagnosis of coeliac disease leaving 13 participants (6 boys, 7 girls). The mean (SD) age of participants was 80 (±38) months. The characteristics, family history and clinical history of these participants are shown in Table 1. Initial clinical assessment of participants showed that a number had symptoms associated with cow’s milk protein allergy (CMPA) or intolerance (CMPI) [13].

**Table 1:** Age, family history and clinical history of 13 participants recruited to Trial 1 and 39 participants recruited to Trial 2.

Characteristics and History	Participants Reporting Characteristic of History			
	Trial 1 (*n* = 13)		Trial 2 (*n* = 39)	
	*n*	%	*n*	%
*Age*				
<3 years	2	15.3	10	25.6
4–6 years	4	30.7	19	48.7
7–9 years	5	38.4	6	15.3
10–12 years	2	15.3	4	10.2
Family history of CMPA or CMPI	4	30.7	7	17.9
Personal history of CMPA or CMPI	0	0	3	7.7
*Physical symptoms of intolerance to cow’s milk*				
Dermatitis, eczema, rhinitis			20	51.3
History of asthma	6	46.1	16	41.0
History of ear infections	9	69.2	9	23.1
Grommets	2	15.3	2	5.1
History of tonsillitis/throat infections	5	38.4	4	10.2
Tonsils removed	1	7.6	2	5.1
Adenoids removed	3	23.0	3	7.7
*Gestation and birth characteristics*				
Maternal thrush during pregnancy	2	15.3	4	10.3
Delivered by caesarian section	3	23.0	8	20.5
Breastfed	6	46.1	12	30.7
Development delayed	3	23.0	11	28.2
*Behavioural issues*				
Consultation with psychologist	2	15.3	6	15.4
Psychosocial disruption of the family	6	46.1	17	43.6
History of recurrent UTI’s	3	23.07	9	23.1
*Constipation symptoms*				
Soiling or encopresis	13	100	25	64.1
Abdominal pain	9	69.2	28	71.8
Anal pain	7	53.8	27	69.2
Anal bleeding	3	23.0	14	35.9
Diagnosis of perianal dermatitis	1	7.6	8	20.5
*Other current symptoms*				
Poor appetite	5	38.4	22	56.4
Nausea or vomiting	5	38.4	8	20.5

Forty participants were recruited to Trial 2, with one excluded from the study due to a subsequent diagnosis of coeliac disease leaving 39 participants, 25 males and 14 females. The mean age of participants was 67 months (±35 months) and the range was 21 to 143 months. The characteristics, family history and clinical history of these participants are also shown in Table 1. Seven participants reported a family history of cow’s milk protein allergy or intolerance. Initial clinical assessment of participants showed that a number had symptoms associated with cow’s milk protein allergy or intolerance, including, asthma, ear infections and grommets [2] but only three of these participants had been diagnosed with cow’s milk protein allergy or intolerance. 

### 3.2. Primary Outcome Measure: Resolution of Constipation

#### 3.2.1. Trial 1

**Figure 3:**
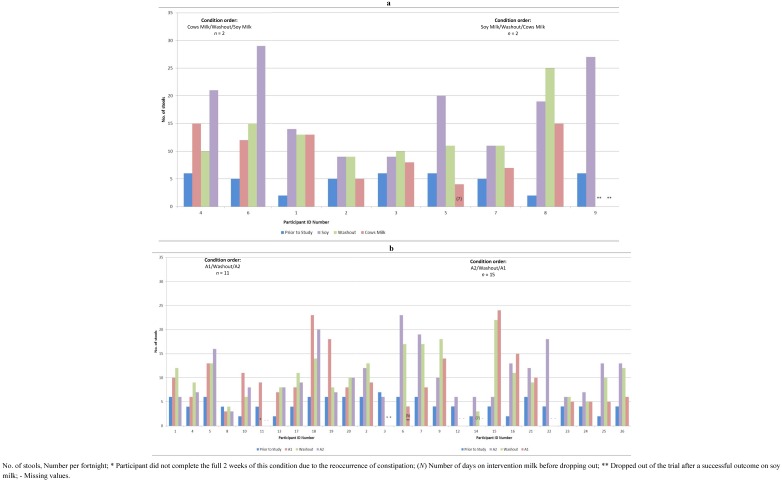
(**a**) Number of stools per fortnight at baseline and on each of the study conditions for Trial 1; (**b**) Number of stools per fortnight at baseline and on each of the study conditions for Trial 2.

Nine of the thirteen participants returned constipation diaries for the study period and eight completed all three dietary epochs. The number of bowel motions per dietary epoch is shown for each participant in Figure 3a.

**Figure 4:**
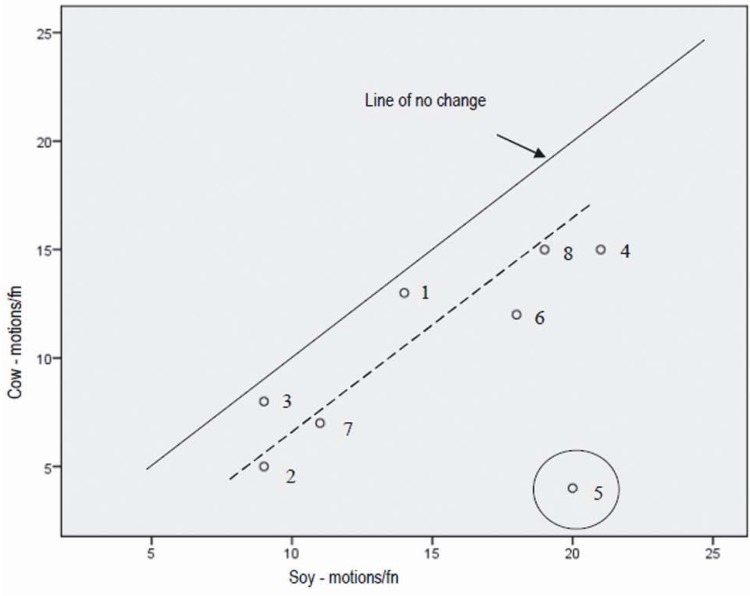
Total number of bowel motions per fortnight during the soy condition *versus* the cow’s milk condition.

The mean number of bowel motions increased from baseline for all participants. No participant experienced constipation (less than 8 bowel motions per fortnight) on the soy milk epoch. During the milk free washout, only one participant experienced constipation and this occurred when the washout followed the cow’s milk epoch. Three participants who showed no constipation on soy milk or washout met the definition for constipation during the cow’s milk epoch. The ninth participant left the study after experiencing resolution of constipation on soy milk in the first epochand could not be included in the analysis. Using the Greenhouse-Geisser adjustment, the differences in mean number of bowel motions between the three epochs (shown in Table 2) was statistically significant, *F* (1.88, 13.1) = 4.58, *p* = 0.03. Follow up statistical analysis, using contrasts and pair wise comparisons and a Repeated Measures ANOVA identified differences in the number of bowel motions per fortnight between the three epochs as shown in Table 3. To further verify the analysis above, the Freidman test for three related variables was applied to the number of bowel motions in each of the three epochs and showed statistical significance (*x*^2^(2) = 9.6, *p* = 0.01). The number of motions for each subject during the cow’s milk versus the soy milk epoch for each participant is plotted below in Figure 4.

**Table 2:** Cases of resolution (greater than 8 bowel motions per fortnight) of constipation and mean bowel motions during a 2 week condition for Trial 1 and Trial 2.

Trial 1						Trial 2				
Bowel Movements and Descriptions	Pre trial ^1^ *n* = 9	Cow’s milk *n* = 8 ^3^	Washout ^2^ *n* = 9 ^3^	Soy Milk *n* = 9	*p*	Pre trial ^1^ *n* = 26	Cow’s milk β casein A1 *n* = 22^ 3^	Washout ^2^ *n* = 23 ^3^	Cow’s milk β casein A2 *n* = 25 ^3^	*p*
Resolution of Constipation *n* (%)	0 (0)	5 (62)	8 (100)	9 (100)	0.01 *	0 (0)	14 (64)	18 (78)	16 (64)	0.12 ^4^
Bowel motions per fortnight M (SD)	5.1 (1.4)	9.9 (4.4)	13.0 (5.2)	15.1 (5.0)	0.03 **	4.42 (1.55)	10.05 (5.75)	10.43 (5.05)	10.56 (5.24)	0.62 ^5^
										0.42 ^6^

* Wilcoxon Signed Rank Sum; ** Paired *t*-test; ^1 ^Reported by participants prior to trial commencement; ^2^ The washout period acted as a condition in itself as it was free from all milk and soy protein; ^3^ The number of participants in each condition is not the same because some participants ceased participation in the study after the various conditions; ^4^ Resolution of constipation = >8 bowel motions per fortnight; ^5^ The difference between the two milk epochs cow’s milk β casein A1 and for cow’s milk casein A2; ^6^ The difference between the 3 epochs.

**Table 3:** Clinical outcomes for participants who completed all 3 conditions of Trial 2: cow’s milk β casein A1 condition compared with the cow’s milk β casein A2 condition, *n* = 21.

	Cow’s Milk β Casein A2			
		Less than 8 motions	More than 8 motions	Row Total
**Cow’s milk β casein A1 **	Less than 8 motions	3 *	6	9
	More than 8 bowel motions	3 **	9	12
	Column Total	6	15	21

*n* = 3 *, <8 on both milks; *n* = 3 **, More than 8 on A1 and less than 8 on A2; *n* = 6, <8 on A1 and >8 on A2; *n* = 9, >8 on A1 and A2.

The line of no change, that is, the point at which the number of motions during the cow’s milk epoch equalled the number of motions during the soy milk epoch is shown in Figure 4. All nine participants experienced more bowel motions in the soy epoch versus the cow’s milk epoch. Participant number 5 had an unusually high number of bowel motions per fortnight during the soy epoch. When this participant was removed from the data set, the results were no longer normally distributed so the tests were repeated using non-parametric analysis with all subjects, with the Repeated Measures ANOVA, (*p* = 0.02). Follow up analyses using the Wilcoxon signed rank tests showed that the significant difference found with the Friedman test was due to the difference between the soy and cow’s milk epochs. This held for both analyses, with and without subject 5. See Table 2 for *p* values.

#### 3.2.2. Trial 2

Twenty-six participants returned constipation diaries for the study period. Thirteen other participants dropped out at various stages of the trial. Reasons included: inability to strictly follow the milk free diet, identified allergy to soy milk during the trial, achieving a resolution on soy milk resulting in withdrawal from the study and death in the family. The rate of constipation resolution (greater than 8 bowel motions per fortnight) and the mean number of bowel motions per fortnight under each epoch for participants are shown in Table 2. The number of bowel motions per dietary epoch is shown for each participant is shown in Figure 3b.

The highest observed resolutions, 18 (78%) participants, occurred during the washout period, when no cow’s milk protein was being consumed. There was no significant difference in the mean number of bowel motions per fortnight for each of the epochs, although there was a tendency to a higher number for the cow’s milk β casein A2 epoch. The mean number of bowel motions increased from baseline for all participants as shown in Table 2.

Table 3 shows twelve participants (57%) experienced more than 8 bowel motions on cow’s milk β casein A1, while fifteen participants (71%) experienced more than 8 bowel motions on cow’s milk β casein A2. However, because each participant participated in both epochs in the study there is more to be understood aboutthe data in Table 3. Examining the results jointly, that is, for the A1 and A2 epochs together, three participants (10%) did not resolve during either the cow’s milk β casein A1 or cow’s milk β casein A2 epochs. Nine participants (43%) resolved under both epochs. Further, six (29%) participants resolved on cow’s milk β casein A2 but did not resolve on cow’s milk β casein A1 (four out of these six commenced on A2). Three (14%) did not resolve on cow’s milk β casein A2, but did resolve on cow’s milk β casein A1 (two out of the three commenced on A1). It is these last two cells that provide the information as to whether A2 differs from A1 and is assessed using the McNemar test for paired categorical data. Based on this analysis, the percentages that resolved on cow’s milk β casein A2, 15 participants (71%) and cow’s milk casein A1, 12 participants (57%), there was no significant difference between the two *p* = 0.51.

The difference between washout and the two milk epochs cow’s milk β casein A1, (*p* = 0.12) and for cow’s milk casein A2, (*p* = 0.62) was not significant. There was a higher percentage resolution on cow’s milk β casein A2 than cow’s milk β casein A1, but the results were not statistically significant. Neither was there statistical significance between the three epochs in motions per fortnight for cow’s milk β casein A1, washout and cow’s milk casein A2, *F* (2, 39.2) = 0.90, *p* = 0.42. As neither of the three epochs was demonstrably different, nor different to washout, it appears that participating in the trial was the most important effect, raising the percentage of constipation resolved (greater than 8 bowel motions per fortnight) from zero prior to the trial to an average level of (57 + 71 + 82)/3 = 70% over the 6 weeks of the study. Secondary outcome measures, such as symptoms associated with constipation on the Bristol Stool Scale were recorded; however, there were no distinguishable trends or patterns between participants (data not shown).

## 4. Discussion

The first trial showed that removal of CMP from the diet of children with CFC significantly increased the number of bowel motions and improved constipation, suggesting that CMP played a role in CFC for these children. All participants in this study experienced resolution (greater than 8 bowel motions per fortnight) of constipation on soy milk compared with the 68% that resolved on soy milk in the study by Iacono and colleagues [3]. The difference may be due to the fact that the Iacono study was not completely CMP free for its duration because the background diet was unrestricted. In addition, the brief period between epochs in Iacono’s study went uncontrolled and the number of bowel motions during this period was not reported. Our study provided a two week CMP free washout period with monitored bowel motions to avoid these limitations. The fact that only three out of eight children in Trial 1 experienced constipation during the cow’s milk epochcould be explained by a trial effect (as discussed below) or by a mixed model of cow’s milk sensitivity, where children with resolution of constipation (greater than 8 bowel motions per fortnight) on this epochmay be exhibiting CMP intolerance rather than CMP allergy. The trial effector Hawthorne effect, (that is, being involved in a study and applying more attention to the problem, may have helped to resolve the problem) possibly occurred in both studies with the overall trend to an increased number of bowel motions from baseline, regardless of epoch. It is possible that consuming a CMP free diet for six weeks may have decreased the total amount of CMP consumed in comparison to participant’s pre-trial diet for some participants (even with the extra 400 mL for the cow’s milk epoch) below a tolerance threshold, or that the requirement to consume 400 mL of fluid in each epoch resulted in an increase in usual total fluid intake (despite being advised to keep total fluid intake stable) and therefore hydration. Future studies should monitor daily fluid intake as a compliance measure (before and during the study). 

Trial 2 was the first experiment of its kind to compare the effects of β casein A1 and β casein A2 moiety on children with CFC. While the mean number of bowel motions increased and some participants reached resolutionof constipation (greater than 8 motions per fortnight) (probably due to the trial effect outlined above), bothepochs reproduced the results of the cow’s milk β casein A1 epoch in Trial 1. It seems that it is not the β casein moiety, in cow’s milk that is causing constipation or if it is, it is not the section that differs between the A1 and A2 variants. Some other component in cow’s milk common to both A1 and A2 milk but not to soy milk may be causing a problem in these susceptible children. Resolution (greater than 8 bowel motions per fortnight) with both cow’s milk β casein A1 and cow’s milk β casein A2 epochs suggests that these children are able to tolerate some CMP before the symptom occurs and possibly 400 mL was insufficient to induce an effect. 

The strengths of this trial were the crossover design with subjects acting as their own controls, the two-week washout period free from cow’s milk and soy milk and their derivatives, and the six week record of bowel outcome measures. A number of limitations have been identified. While a double blind protocol was followed, it is unlikely that it was achieved in Trial 1 due to the easily identifiable taste and smell of soy milk. This may have had a positive psychological effect on some participants’ constipation if they believed cow’s milk to be the cause. This was not the case in Trail 2 given that A1 and A2 milk are identical in appearance, flavor and smell. The assignment of participants while even in those recruited, was not even in the final participant population, due to a bias where those who commenced on cow’s milk being more likely to drop out of the study.

The study had a high participant burden which may have contributed to lack of completion in both trials. Nutrition intervention studies are notorious for the challenge in retention caused by the burden of dietary change [14], resulting in relatively small numbers of completers. The rigors of a dietary trial based on exclusion of a nutrient, CMP, commonly found not only in its most obvious dairy sources but also hidden in many processed foods in Australia proved to be difficult for many participants. Further burden was placed on participants by asking them to collect data regarding their or their child’s bowel motions on a daily basis. While the families who ceased participation were not formally interviewed, some reported reasons for dropout including: death in the family, trial length, participating only to obtain results of the baseline blood test; and an allergic reaction to soy milk (reported by one participant). For at least one participant, achieving an improved bowel function on soy milk resulted in a withdrawal from the study after the first epochso that the commencing milk affected the research process, if not the results. 

In any further research investigations, CFC should be clearly and commonly defined. Replications of this study should commence the protocol with a two-week washout period. Bowel motions should be recorded and reported during this time. Higher volumes of cow’s milk need to be consumed to determine the threshold that causes constipation in CMPI and actual milk and other fluid consumption needs to be recorded daily. Participants should be well monitored to assist with compliance and increase retention. Investigations into the immunological or biochemical mechanism occurring in CFC are required, including investigations of the intolerance reactions and how they affect nerves in the gastrointestinal tract. 

## 5. Conclusions

The results of Trial 1 demonstrate an association between CFC and cow’s milk consumption while trial 2 failed to show an effect from type of casein. Some other component in cow’s milk common to both A1 and A2 milk may be causing a problem in these susceptible children. Investigations into the immunological or biochemical mechanism occurring in CFC are required, including investigations of the intolerance reactions and how they affect nerves in the gastrointestinal tract.
